# Systematically searching for and assessing the literature for self-management of chronic pain: a lay users’ perspective

**DOI:** 10.1186/1471-2318-14-86

**Published:** 2014-07-27

**Authors:** Pat Schofield, Blair H Smith, Denis Martin, Derek Jones, Amanda Clarke, Paul McNamee, Ron Marsh, Michael Morrison, Rosemary Morrison, Sheena Fowler, Geraldine Anthony, Carrie Stewart

**Affiliations:** 1 University of Greenwich, School of Health & Social Care, Avery Hill Campus Eltham, London SE9 2UG, UK; 2 Division of Population Health Sciences, University of Dundee, Dundee, UK; 3 University of Teesside, Tees Valley, UK; 4 Northumbria University, Newcastle, UK; 5 University of Aberdeen, Aberdeen, UK; 6 Aberdeen (Older adults with an interest in pain management), Aberdeen, UK

**Keywords:** Older adults, Literature for self-management of chronic pain, Strategies for managing chronic pain, Involving older adults in research

## Abstract

**Background:**

The Engaging with older adults in the development of strategies for the self management of chronic pain (EOPIC) study aims to design and develop self management strategies to enable older adults to manage their own pain. Involving older adults in research into chronic pain management will better enable the identification and development of strategies that are more appropriate for their use, but how can perspectives really be utilised to the best possible outcomes?

**Method:**

Seven older adults were recruited through a local advertising campaign to take part. We also invited participants from the local pain services, individuals who had been involved in earlier phase of the EOPIC study and a previous ESRC funded project. The group undertook library training and research skills training to facilitate searching of the literature and identified sources of material. A grading tool was developed using perceived essential criteria identified by the older adults and material was graded according to the criteria within this scale.

**Results:**

Fifty-seven resources from over twenty-eight sources were identified. These materials were identified as being easily accessible, readable and relevant. Many of the web based materials were not always easy to find or readily available so they were excluded by the participants. All but one were UK based. Forty-four items were identified as meeting the key criteria for inclusion in the study. This included five key categories as follows; books, internet, magazines, leaflets, CD’s/Tapes.

**Conclusion:**

This project was able to identify a number of exemplars of self management material along with some general rules regarding the categories identified. We must point out that the materials identified were not age specific, were often locally developed and would need to be adapted to older adults with chronic pain. For copyright issues we have not included them in this paper. The key message is really related to the format rather than the content. However, the group acknowledge that these may vary according to the requirements of each individual older adult and therefore recommend the development of a leaflet to help others in their search for resources. This leaflet has been developed as part of Phase IV of the EOPIC study.

## Background

Chronic pain is highly prevalent amongst older adults, with at least 50% of community-dwelling older adults reporting symptoms of pain lasting longer than three months [1-3]. The management of chronic pain is complex. The condition involves both physical and psychological experiences [4] and its trajectory and prognosis can vary widely, resulting in numerous debilitating psychosocial and functional consequences [5-10]. Self-management, the daily participation of patients to manage and control their symptoms, is a recommended step in the management of long term conditions [11-14]. Active management of pain symptoms has demonstrated reduced pain severity, improved psychological wellbeing and increased quality of life [15-17]. Recently, attention has turned to exploring novel methods of delivering self-management advice to older adults with pain which can address the complexities involved in managing this condition (for example, see the Engaging with older adults in the development of strategies for the self management of chronic pain EOPIC Programme, through which this study was conducted, (http://eopic@dundee.ac.uk).

Consumer consultation in health care has evolved since the 1990’s and patient participation in the design and development of health care research is increasing [18]. Service users can be involved in many aspects of study design and delivery and INVOLVE [19] provide useful guidance for researchers who wish to involve the public in their research. They define user involvement as doing research ***with*** or ***by*** the public rather than ***to, about***, or ***for*** the public. (INVOLVE pg II). Participatory research aims to encompass the needs of the participants in a way that challenges the traditional researched/researcher dichotomy. It requires the researcher to create an environment which supports negotiation, mutuality and respect between service users and researchers [20]. Power and role responsibilities must be distributed equally between users and researchers [20]. Despite policy developments and changes in attitudes towards how we conduct research, research has still been criticised for being carried out “on” participants rather than “with” participants [21]*.* For example Johnson & Abbott in 1999 [22] looked at a study of continuing health care policies and were surprised to find that many carers and older people were unaware that they had even been consulted. Another study identified that older people often had their choices made by others on their behalf and the choices made were actually influenced by financial resources instead of consumer opinions [23]. Nevertheless, numerous benefits and successes of engaging service users’ experiences in setting research agendas and assisting with the conduction of research, specifically systematic reviews and the development of self-help approaches, have been described previously [24,25,20].

As service users are central to self-management, feedback from service users about current self-management materials is as important to improving materials as the development of new approaches [25]. Service users also bring specialist experience and can play active roles in defining the scope, locating and appraising the literature, interpreting findings and writing up of reports [24,20]. This present study sought to collaborate with older adults to review existing pain self-management materials, devise a set of guidelines, in close adherence to Scottish Intercollegiate Guidelines Network principles (http://sign.ac.uk/methodology/index.html) and guide the development of future pain self-management resources designed for older adults.

## Methods

### Design

This lay-user review of older adults’ pain self-management materials used a co-action approach which allows users and researchers to share their knowledge and experiences to create a new understanding and learn together [26]. Our aim was to establish a group of lay people and researchers who would work together to achieve the following objectives:

•Identify older adults’ pain self-management material available,

•Develop a system of grading the quality of these materials,

•Identify exemplars of pain self-management materials, and

•Formulate a set of recommendations to guide future development of these materials.

### Review group

Seven older adults with an interest in pain management and self help were identified and invited to take part in the project. These adults were recruited through a local advertising campaign which invited older adults to take part in the research. Our experience through recruiting via the local press had been successful previously. We also invited participants from the local pain services, using our contacts within the pain team. Anyone who was over the age of 65 was invited to take part and if they had experience of chronic pain. We also invited individuals who had been involved in an earlier phase of the EOPIC study as participants, members of the EOPIC advisory group and a previous ESRC funded project. One of the PhD students (CS) linked to the study and the Chief Investigator (PS) were also involved. Library training was provided to the whole group, which involved developing internet and literature searching skills and support in accessing self help material and grey literature. We also provided a training programme in basic research skills which included an understanding of research terminology, methods, reading literature and methods of critical appraisal. This was provided by the information scientist on the local site.

#### Ethical consideration

The study was approved by the North of Scotland Research Ethics Committee (09/SO802/93). All participants were given an information sheet explaining the purpose of this work and a consent form to sign. Verbal and written informed consent was obtained from all participants before they took part in this study. Opportunity was given to ask questions or withdraw approval at any stage.

### Scope & search strategy

Scope was determined through group discussion and the consensus was to consider a wide range of formats of materials. The following sources for locating self-management advice resources were identified:

Carers Centre, chemist, library, CKS NHS, Arthritis Research Council, Diabetes UK, INTUTE, Scirus extras. Pub Med, Web MD, British Pain Society, American Pain Society, Australian Pain Society, Pain Concern, NHS e-library, SIGN, NICE, Age UK, Local GP Practices, Patient UK, Pain Association Scotland, Strategic Health Authorities, Health Boards, Boots Web, Tapes, CD’s and Books, periodical magazines.

Simple Google searches were performed which resulted in over 29 million sources which was completely un-manageable, so we focussed upon specific data bases, such as NHS Evidence, NHS e-library, Boots evidence, NICE NHS information and Web MD. These were recommended by the information scientist as they are accessible to the general public and therefore represented the data bases that would be most likely accessed by the public in their own homes recommended library resources such as INTUTE etc. were proposed by the Information Scientist.

### Identification of relevant items

For the purpose of the EOPIC study we defined self management as; “*a single approach or combination of approaches that can be initially taught by any health professional or learned by an individual to enable them to minimise the impact their chronic pain can have on everyday life”.* This definition was based upon the advice of our service user group and advisory group of professionals for the EOPIC study*.* Each group member was allocated one or more of the sources detailed above and asked to identify self-management materials from that source. The identification criterion of relevant materials was that the items must be self-management materials relevant to adults or older adults with non-malignant chronic musculo-skeletal pain.

Once all materials were identified, the group discussed the materials they had found, categorised each item into its appropriate format (books/ e-books, internet sites, magazines, leaflets, CD’s/tapes), and began to develop criteria to determine the quality of the items. Monthly meetings took place to discuss the process and offer any support.

### Grading system

The sharing of experiences from the process of searching for the literature, general observations about the quality of identified materials, and personal experiences using self-management materials, helped the group develop a set of criteria (a grading system 0–10) by which they wished to assess the quality of each item. This grading system outlines the desirable characteristics of acceptable self management literature identified by lay persons in the study (Table 1 below). In order to develop the grading system, participants were given access to the already developed grading systems (CASP (http://www.casp-uk.net/ accessed 10th June 2014), SIGN http://www.sign.ac.uk/pdf/gradeprincipals.pdf accessed 10th June 2014). As part of their research training, they had been given a session on grading material. The grading systems were supplied to help them gain an understanding of the system. But participants were keen to develop a system that was relevant to them; older people with chronic pain. The grading system is comprised of ten items; each item could be scored as either 0 (does not demonstrate this feature) or 1 (demonstrates an acceptable level of this feature). A total score for each material reviewed was calculated (0–10). Any material with an overall score of <6 was rejected.

**Table 1:** Grading system developed for the study

	**1st Reviewer**	**2nd Reviewer**
Colour/Attractiveness		
Print size/font		
Succinct information		
Accessibility (web, print, range, address, phone number)		
Contact details of author		
Registration process for updates		
Musculo-skeletal pain and relevance		
Treatment 9Traditional/Complementary		
Funded by		
Does it offer a network of support		
Any other comments		
**Total score**		
**Total agreed score**		

### Data extraction process

Each group member was allocated to one of the categories of material identified and reviewed the material using the grading system. Members then swapped categories so that all items would be reviewed by a second member of the group as per SIGN recommendations. Each of the lay members of the group worked with a professional member to ensure that they had support and guidance if needed. Any disagreements between reviewers’ scores for items were resolved by discussing within the main group to obtain an agreed score. The group agreed that any material with a score less than 5 would not be included in the final review.

### Analysis

The final review to identify exemplars and formulate a set of recommendations was conducted through group discussion. Decisions made as to which materials would be considered exemplars and specific recommendations to make were made through gaining a consensus agreement of all members.

## Results

The group identified 57 individual self-management materials from 28 sources. Forty-four items were identified as meeting the key criteria for inclusion and scored ≥5 (detailed in Table 2). Common reasons for resources being rejected were: the absence of a registration process for updates, no provision of support network details, no disclosure of the funding body and poor graphic design of materials (i.e. not attractive to look at, low readability). The group could only identify one resource specifically developed for the self management of chronic pain in older adults.

**Table 2:** Self-management literature reviewed

**Source**	**Document**
*Book*	
International Association for the Study of Pain (IASP)	Pain management for older adults- Self help guide
	Hadjistavropoulos T, Hadjistavropoulos H, eds. Pain Management for Older Adults: A Self-Help Guide. Seattle: IASP Press, 2008. ISBN 0-9310-9270-1; (Available from: http://ebooks.iasp-pain.org/pain_management_for_older_adults)
*Leaflets or PDF Download Files*	
British Pain Society (BPS) http://www.britishpainsociety.org/	Managing Cancer Pain
	Managing your pain effectively using OTC medicines
	Pain and problem drug use
	Intrathecal Drug Delivery Systems
	Pain Management Programmes
	Stimulating Spinal Cord
	Using meds beyond licence
	Opioids for persistent pain
	Understanding & managing pain
Scottish Intercollegiate Guidelines Network (SIGN) http://www.sign.ac.uk/	Cancer Pain
NHS Fife https://sites.google.com/site/fifepaininfo/home/for-patients	Managing your pain (physio foot notes 3& 6
	Bums off seats
CKS NHS http://cks.nice.org.uk/	Pain and Arthritis Information Booklet
Patients Association http://www.patients-association.com/	Pain- A hidden problem
Pain Concern http://painconcern.org.uk/	Factsheet
	Flare up planning
	Good days and bad days
	Improving your posture
	Keeping flare ups at bay
	Pain management: A new lease of life
	Travelling abroad
	Nerve pain
	Enjoying outings
	When your pain management skills seem to have stopped working
	Wakey wakey…
Pain Association Scotland http://www.painassociation.com/	Managing chronic pain in 10 easy steps
The Pain Toolkit http://www.paintoolkit.org/	Calendar 2011
	The self-care tool kit
North Bristol NHS Trust	Strategies for keeping mobile
	Pain Medication General Information
NHS Direct http://www.nhsdirect.nhs.uk/	Help & advice for living well with a LTC
NHS Kirklees	Chronic Pain
Action on Pain http://www.action-on-pain.co.uk/	Create a pain plan
Long Term Condition Alliance	My condition, my terms, my life
http://www.alliance-scotland.org.uk/	
NHS QIS	Improving services for people with chronic pain
	http://www.healthcareimprovementscotland.org/previous_resources/process_documentation/nhs_qis__nice_advice.aspx
Useful Websites & Webpage’s	
Bupa	http://www.bupa.co.uk
	This site has a range of patient information resources
Pain Relief Foundation	http://www.painrelieffoundation.org.uk
Pain Support	http://www.painsupport.co.uk
CD’s, Videos, MP3’s & Magazines	
Neil Berry CD http://www.paincd.org.uk/	
Able Radio http://www.ableradio.com/	
Newsletter: Pain Matters http://www.painconcern.org.uk/how-we-help/pain-matters-magazine/	
Newsletter: Pain Support http://www.painsupport.co.uk/	
Womens Weekly/Top Sante etc.	

### Books

A range of books were identified and reviewed but few included sections in relation to older adults and only one was included the final review. The group identified that the size of font, the attractiveness of the book (i.e. colour, illustrations) and the readability (“easy reading”) were important indicators of usefulness of books. Preferred books provided the right level of information; a broad coverage and not too detailed. The group concluded that an interactive / diary type book which met all the criteria used within this review would be most useful. The lay members particularly liked the “telling children” range of books and would like to see something similar developed about chronic pain.

### Internet

The group consensus was that internet sites need to come with *a health warning*. They considered the internet to provide good sources of information, but were concerned about how up to date information was and the authenticity of their content. There were also concerns raised about how results can vary according to the search terms used, reflecting how individuals may define and understand pain and its management differently. Some websites were difficult to navigate and were confusing to use, and ability to seek advice from these sites may depend on the individual’s internet skills. The group confirmed this finding to include NHS sponsored sites. Accessibility of the internet was also questioned as the group had a concern that not everyone has access to the internet.

### Magazines/periodicals

A number of health and general well-being themed magazines were reviewed. The group did identify a couple of pain management specific articles, but did not identify any magazines which had regular pain advice sections. Concerns raised about this type of material were in knowing how up to date the advice provided was, and that advice was often given by generalists (i.e. health journalists) as opposed to specialists (qualified health professionals).

### Leaflets

This category of materials was by far the largest, with a substantial number of leaflets reviewed. The group were able to identify three good exemplars of pain self-management advice provided by Pain Concern, Pain Association Scotland, and the NHS sponsored Pain Toolkit. These exemplars impressed the group with their attractiveness (bright colours), readability (easy to read), concise and relevant information. The group also agreed that these leaflets could be used by anyone regardless of age.

### CD’s & tapes

The group concluded that these materials were typically quite general and not particularly specific to chronic pain. However, their use for certain strategies, such as relaxation, may be beneficial for some individuals.

### Group recommendations

Overall, leaflets seemed to be the preferred format but in addition there are a few good internet sites and some useful books. The consensus opinion of the group was that a range of formats is probably the best way of ensuring the specific needs of individuals are met. Whilst some items were identified as being the most useful, it must be remembered that this is largely personal choice and the group would not wish to recommend any item over and above others. No material covered a topic entirely and so the group recommends readers select the most appropriate material for their personal needs and to consider further reading. The group also recommends the development of a guide for reviewing self help material that may enable users to search and review material for themselves.

## Discussion

To the best of our knowledge, this is the first review of pain self-management materials conducted through a collaboration of researchers and lay older adults with chronic pain. The collaborative approach undertaken combines the strengths of scientific rigour with the personal experiences and perspectives of the key stakeholders. We developed a method of assessing the quality of these materials from the lay users’ perspective. We were also able to identify exemplars of pain self-management materials and make recommendations about the desirable qualities of these materials. Our results provide those who seek to develop or refine pain self-management materials insights as to how their materials are, or may be, perceived by those who will use them.

In terms of recommendations, there were a number of exemplars and formats that were considered to be appropriate and met all or most of the ten criteria as developed by the group. It is interesting to note that in both the criteria and recommendations, the delivery of these materials (i.e. mode, design) and range of choices available appeared to be of greater importance than the actual content, so long as content was from a reliable source. This review also uncovered a need for the development of a user guide to locating pain self-management materials. Our findings resonate with those of Lucock *et al.* study in 2007 [25] who also found a desire for choice of format and a guide to accessing self-help resources amongst mental health service users. It is envisaged that a guide enabling users to search and review material for themselves will be developed as a result of this study.

Whilst we have made efforts to empower the user members of the group and create equality between service user and researcher members, it is important to discuss the impact of researcher’s involvement [26]. Differences in motivations between researchers and service users [27] and pressures upon researchers to conform to some level of academic priorities [20] need to be overcome. Additionally, removing any perceived power structure within the group is important [27]. Therefore both researchers and service users undertook the same training from the information scientist. This learning together acted as a method of promoting power equality between researchers and service users. It provided a level playing field in terms of knowledge as to how to conduct this project.

Further difficulties with this approach include time pressures, resource limitations and establishing group dynamics [24]. This said, we found that when researchers were motivated and enthusiastic about public participation, and where funding for such an initiative was available, we had few difficulties in achieving an ideal environment to foster this approach. Funding for this project was built into a grant application for the EOPIC study, awarded by the Medical Research Council (MRC). We acknowledge that whilst the MRC are very supportive of public involvement initiatives such as this review, though some other funding bodies may not always attach such a high priority. In terms of time and resources used, this project took around one year to complete. Our main financial costs included participants’ travel expenses to attend meetings, and the provision of information skill training.

## Conclusion

Though this review was unable to identify any pain self-management materials specifically targeted at older adults needs, other than the book developed in the US. The book was considered useful, but the participants felt that it presented a challenge in terms of font size for easy reading and they commented that the book was specifically targeted at a US population which was not really relevant for their needs as older adults in the UK. Furthermore, they wanted resources that were short, easy to read and quick to access specific aspects relevant to their circumstances in the UK. The identification of examples of good practice resulted in a set of user-developed recommendations to be taken forward in future development of these materials. It is important to note here that many of the examples of good practice were UK based materials which the users considered important for culture specific reasons and in terms of literacy levels of some of the participants. Whilst the bias may be towards UK based resources. This is no surprise as the study was taken from the perspective of the older adults who are UK based. The most important recommendation is that of choice and flexibility; offering self-management materials in a variety of formats which can meet the diverse range of needs of those who will use them, allowing individuals to decide which method best suits their own needs were important, hence the group’s reluctance to recommend any of the materials over and above others. We have demonstrated that this collaborative approach is both useful and feasible in this setting providing support for future participatory research in this area. The EOPIC study will enable this collaboration to go forward.

## Competing interests

The authors declare that they have no competing interests.

## Authors’ contributions

PS, BS, DM, DJ, AC, PM, GA, CS all made substantial contributions to the conception and design of the study. PS &CS carried out most of the data collection and analysis of the data. RM, MM, RM &SF were study participants who were trained in research skills and literature searching. BA helped with analysis and drafting the manuscript. All authors contributed to data analysis and interpretation, critically commented on, and approved the final manuscript.

## Pre-publication history

The pre-publication history for this paper can be accessed here:

http://www.biomedcentral.com/1471-2318/14/86/prepub
